# Comprehensive Management of Oroantral Fistula Secondary to Chronic Maxillary Osteomyelitis: A Case Report

**DOI:** 10.7759/cureus.95120

**Published:** 2025-10-22

**Authors:** Gursimrat K Brar, Asmita Sharma, Asmita Sodhi, Deepinder Sodhi, Parttyancha Bansal

**Affiliations:** 1 Department of Oral and Maxillofacial Surgery, Dasmesh Institute of Research and Dental Sciences, Faridkot, IND; 2 Department of Prosthodontics, Dasmesh Institute of Research and Dental Sciences, Faridkot, IND; 3 Department of Otolaryngology, Guru Gobind Singh Medical College and Hospital, Faridkot, IND

**Keywords:** chronic osteomyelitis, comorbidities, maxilla, oroantral fistula, systemic

## Abstract

This case report describes the successful management of a rare oroantral fistula (OAF) secondary to chronic osteomyelitis in a 50-year-old woman presenting with persistent right maxillary swelling, purulent discharge, and oroantral communication persisting for six months. The case was complicated by systemic comorbidities and increased susceptibility to infection, with no history of dental extractions or trauma. The initial misdiagnosis of nasal polyps led to an unsuccessful endoscopic sinus surgery. Cone-beam computed tomography revealed extensive osteolytic lesions and buccal bone necrosis, whereas histopathology confirmed chronic osteomyelitis with hypertrophic sinus mucosa. Surgical intervention under general anesthesia involved atraumatic extraction of the affected teeth, debridement of the necrotic bone, excision of the hypertrophic sinus lining, and closure with a buccal advancement flap. Postoperative care included antibiotics and medical optimization of comorbidities, resulting in complete symptom resolution at the one-month follow-up. This case highlights the diagnostic challenges of osteomyelitis-driven OAF and emphasizes the critical role of comprehensive imaging, histopathological confirmation, and a multidisciplinary approach that integrates surgical precision with systemic management of the disease. These successful outcomes underscore the need for tailored strategies to address complex OAFs, particularly in patients with systemic risk factors, to achieve optimal healing and prevent recurrence.

## Introduction

An oroantral fistula (OAF) is a pathological communication between the oral cavity and maxillary sinus, often arising as a complication of dental extractions in the posterior maxilla, where the roots of the premolars and molars are anatomically close to the sinus floor [1]. Other etiologies include dentoalveolar infections, maxillofacial trauma, pathological lesions, chronic osteomyelitis, and complications of dental implant procedures [2]. Chronic osteomyelitis, characterized by persistent bone inflammation and necrosis, can exacerbate OAF formation by compromising the integrity of the sinus floor, particularly in cases involving the first and second maxillary molars due to their proximity to the sinus [3]. This condition presents significant clinical challenges, with symptoms such as fluid regurgitation into the nasal cavity, altered speech, and recurrent sinusitis, severely affecting the patient’s quality of life [4].

Untreated OAFs associated with chronic osteomyelitis can lead to persistent maxillary sinusitis, mucosal thickening, and extensive bone necrosis, resulting in chronic inflammation [3,5]. The literature suggests that 50%-60% of untreated OAF cases progress to chronic sinusitis, with hypertrophic changes in the sinus mucosa being a common finding [4]. The bidirectional exchange of oral bacteria and nasal secretions further aggravates this inflammatory process, necessitating prompt diagnosis and intervention. Clinical and radiographic evaluations, particularly with cone-beam computed tomography (CBCT), are essential for assessing defect size, bony architecture, and associated sinus pathology [4,6]. CBCT is the preferred imaging modality because of its detailed visualization and lower radiation exposure compared with conventional computed tomography scans [7].

Treatment strategies depend on the defect size, complexity, and underlying pathology, ranging from spontaneous healing for defects < 2 mm to surgical interventions for larger communications, especially when complicated by chronic osteomyelitis and sinus pathology [8]. Surgical options include local flaps, grafts, and advanced techniques such as endoscopic sinus surgery [9,10]. This case report details the management of an OAF associated with chronic osteomyelitis, buccal bone necrosis, and hypertrophic sinus lining in a 50-year-old woman and highlights the importance of comprehensive diagnosis and tailored surgical strategies to achieve successful outcomes.

## Case presentation

A 50-year-old woman presented to the outpatient Department of Oral and Maxillofacial Surgery at Dasmesh Institute of Research and Dental Sciences in Faridkot, Punjab, India, with symptoms localized to the right maxillary posterior region near the maxillary sinus. She reported a six-month history of insidious facial swelling and purulent discharge, initially managed by an Ear, Nose, and Throat (ENT) specialist with functional endoscopic sinus surgery (FESS). Despite this intervention, the symptoms persisted and worsened, leading to her referral to the Department of Oral and Maxillofacial Surgery. The patient reported chronic discomfort and functional impairment without acute exacerbations.

**Figure 1:**
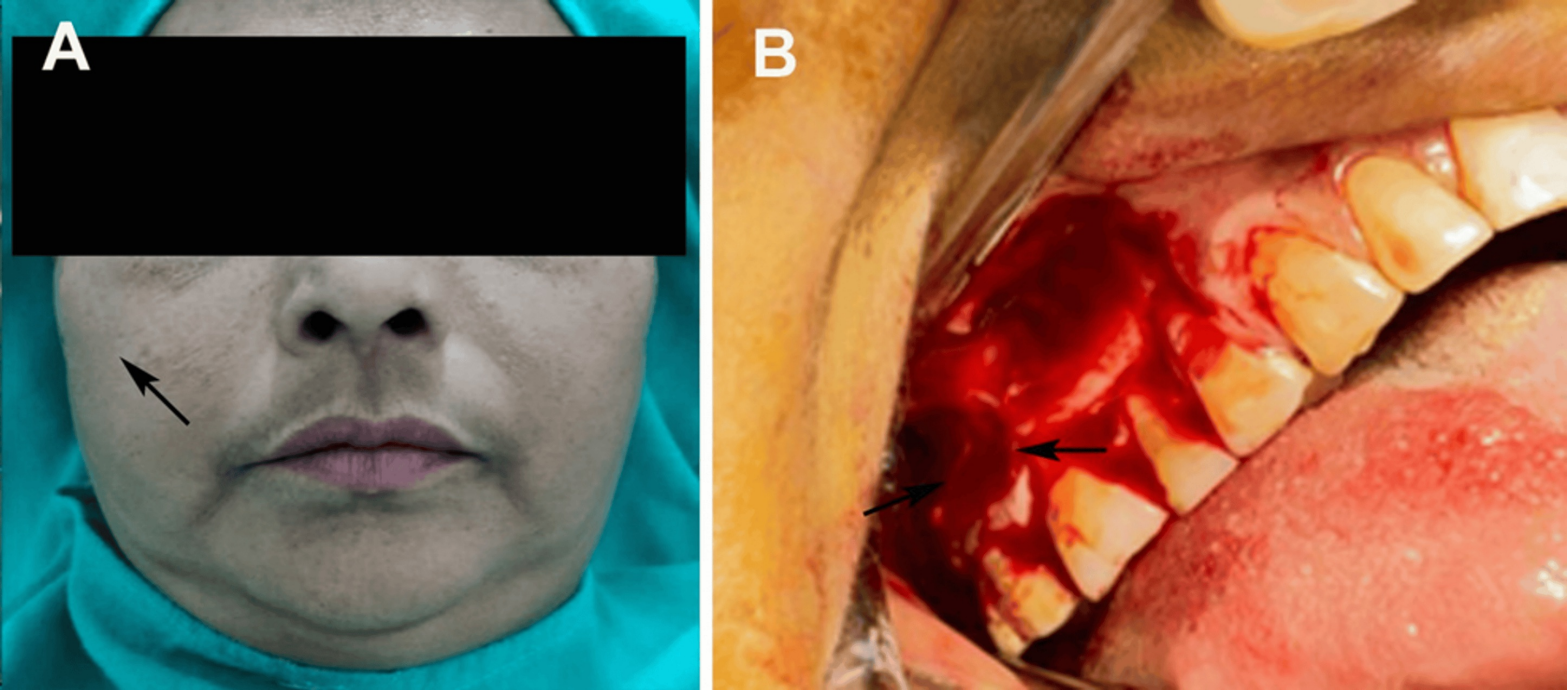
(A) Extraoral examination revealed mild swelling of the right side of the face; (B) intraoral examination revealed a sinus opening at the buccal plate of the maxillary second molar tooth. Original images used with the patient's consent for publication.

Her medical history included controlled type 2 diabetes mellitus, which likely contributed to impaired healing and infection susceptibility, and a four-year history of hypertension managed with unspecified medications with unclear compliance and uncontrolled status. No significant dental history, prior extractions, trauma, or maxillofacial surgeries were reported for the affected region. The patient’s social history, including tobacco or alcohol use, was unremarkable. Her chief complaint was persistent mild swelling and purulent discharge from the right maxillary posterior region, described as diffuse, located in the middle third of the face, extending superoinferiorly approximately 2 cm from the inferior orbital margin to a line joining the corner of the mouth, and anteroposteriorly 2 cm from the ala of the nose to the anterior tragus of the ear, with pain rated 3/10 on a visual analog scale and mild edema (Grade 1 on a 0-3 scale). Intraorally, an OAF was identified with a 4 mm diameter and 6 mm tract length, confirmed via probing, with purulent discharge from the buccal aspect of tooth 17. Dental examination revealed tooth 17 with Grade 1 mobility, probing depths of 6 mm (mesial), 7 mm (distal), 5 mm (buccal), and 5 mm (palatal), non-vital on electric pulp testing, and periapical radiolucency on CBCT, indicating chronic apical periodontitis with mild periodontal bone loss. Teeth 16 and 18 showed probing depths of 4-5 mm, were vital, and had no periapical pathology but were near the osteolytic lesion (Figure 1).

**Figure 2:**
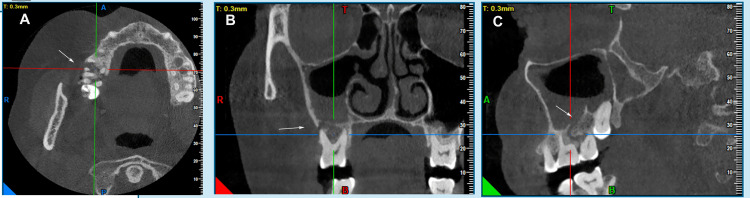
Cone-beam computed tomography (CBCT) of the maxilla showing opacification in the region of the second molar and a soft tissue shadow in the maxillary sinus on the right side: (A) axial view; (B) coronal view; (C) sagittal view. Original images of the patient, used with the patient's permission.

Extraoral examination revealed tender swelling with a mildly elevated skin temperature, indicating underlying inflammation or infection, but no cervical lymphadenopathy, suggesting localized pathology. Routine laboratory investigations, including complete blood count (CBC), bleeding time (BT), coagulation time (CT), and random blood sugar (RBS), were within normal limits despite a history of diabetes, suggesting recent disease stabilization. The viral markers were non-reactive, electrocardiography (ECG) showed a normal sinus rhythm, and chest radiography was unremarkable. Radiographic evaluation via the paranasal sinus (PNS) view showed radiopacity in the right maxillary sinus, whereas CBCT revealed a 2.5 cm × 1.8 cm × 1.5 cm osteolytic lesion involving the right posterior maxilla and maxillary sinus, with a 1.2 cm × 0.8 cm buccal bone necrosis defect, loss of trabecular architecture, and erosion of sinus walls, indicating a destructive process beyond simple sinusitis (Figure 2).

Based on the clinical presentation, radiographic findings, and medical history, several differential diagnoses were considered. Chronic maxillary sinusitis with nasal polyps was suggested by the patient’s history of FESS, persistent sinus symptoms, and radiopacity on the PNS view. However, the osteolytic lesion and buccal communication were atypical features. Odontogenic sinusitis was possible due to purulent discharge near tooth 17 and the proximity of the maxillary molars to the sinus; however, the absence of prior dental procedures and extensive bone destruction made this diagnosis less likely. A benign or malignant neoplasm, such as squamous cell carcinoma or an odontogenic cyst, was considered because of the osteolytic lesion; however, the lack of systemic symptoms, lymphadenopathy, or aggressive growth patterns reduced this possibility. Chronic osteomyelitis with OAF is strongly supported by buccal bone necrosis, sinus communication, hypertrophic sinus lining, and uncontrolled diabetes as risk factors. Fungal sinusitis is considered an immunocompromised state, but the absence of characteristic fungal elements on imaging makes it improbable.

**Figure 3:**
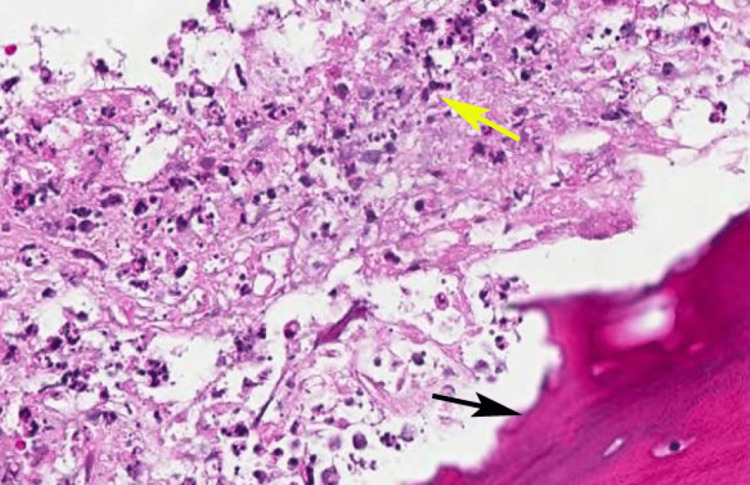
Histopathology of the lesion showing a sequestrum of bone (black arrow) and chronic inflammatory cells (yellow arrow). Hematoxylin and eosin-stained section at 40× magnification. Original image of the patient's sample, used with the patient's permission.

Surgical exploration was performed to confirm the diagnosis. Intraoperative findings revealed necrotic bone (sequestra), hypertrophic sinus tissue, and clear oroantral communication. Tissue samples, including necrotic bone and sinus mucosa, were sent for histopathological examination. Histopathological examination confirmed chronic osteomyelitis, characterized by inflammatory cell infiltration, bone necrosis, and sequestrum formation, along with hypertrophic changes in the sinus mucosa. No evidence of malignancy or fungal infection was observed. These findings, combined with clinical and radiographic evidence, established a definitive diagnosis of chronic osteomyelitis of the maxillary bone, leading to OAF with associated buccal bone necrosis and a hypertrophic sinus lining (Figure 3).

**Figure 4:**
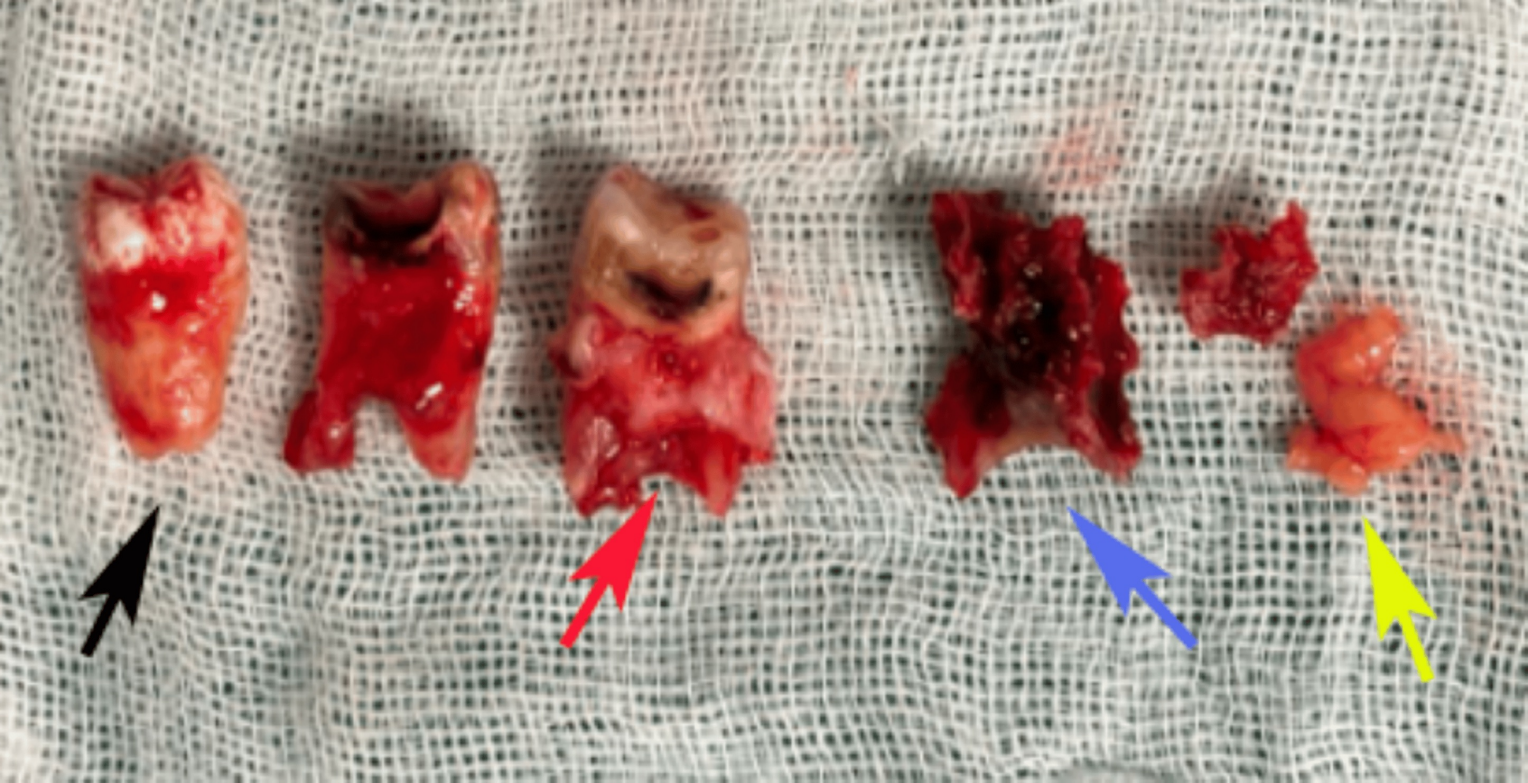
Surgical excision of the lesion and removal of molar teeth (black arrow), infected tissue attached to the root of the molar tooth (red arrow), sinus lining (purple arrow), and sequestrum of bone (yellow arrow). Original images of the patient's sample, used with the patient's permission.

Surgical intervention was planned under general anesthesia induced with nitrous oxide and intravenous propofol, with oral intubation using a 6.5 mm endotracheal tube for controlled ventilation. Preoperative antibiotics were administered to mitigate infection risks given the patient’s diabetic status, and sterile protocols were followed. A crevicular incision was made from tooth 15 (right maxillary second premolar) to tooth 18 (right maxillary third molar), with an anterior-releasing incision at the mesial aspect of tooth 15 for adequate flap mobility. A full-thickness mucoperiosteal flap was elevated to expose the buccal bone necrosis and OAF tract while preserving the periosteal vascularity. Teeth from the right maxillary first molar to third molar (16, 17, and 18) were atraumatically extracted because of their involvement in the infection process and mobility. Necrotic bone sequestra were curetted, and the hypertrophic sinus lining was excised to eliminate the infection nidus and prevent recurrent sinusitis (Figure 4).

**Figure 5:**
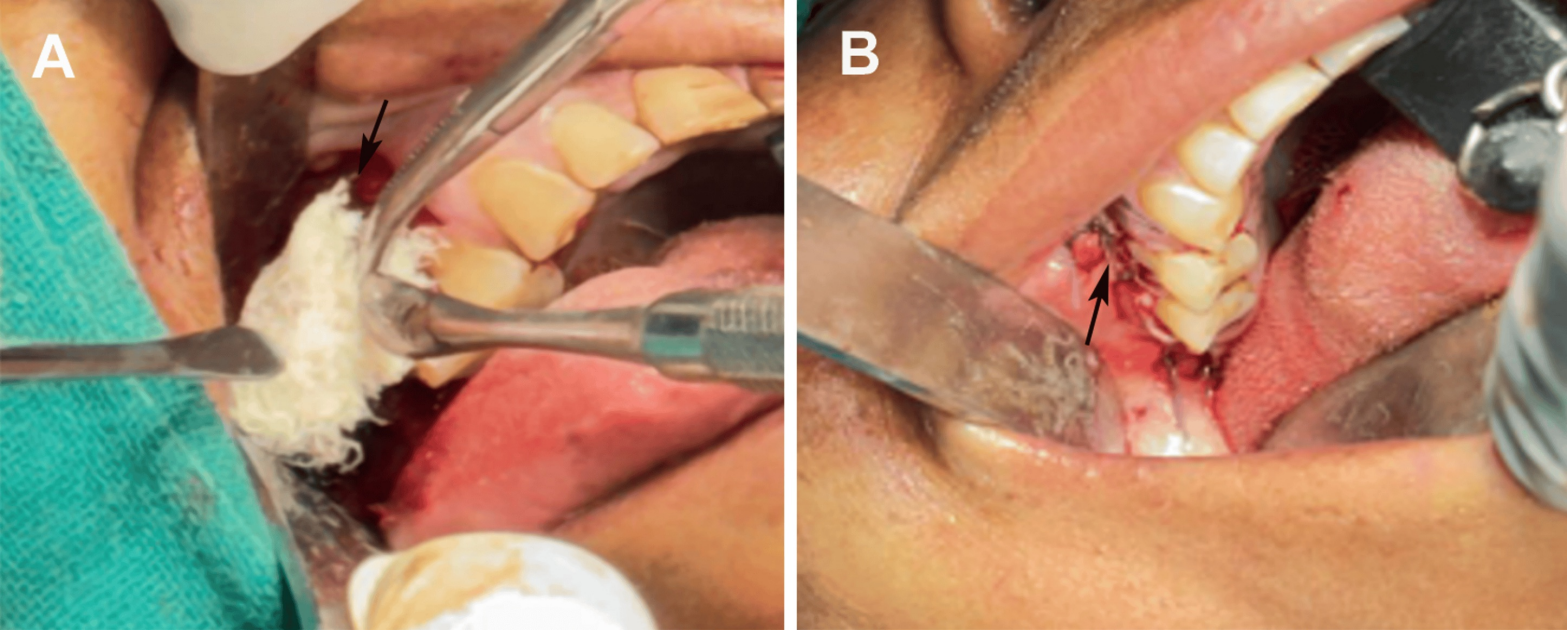
(A) Hemostasis was achieved with an absorbable hemostatic agent; (B) placement of sutures. Original images of the patient, used with the patient's permission.

The surgical site was irrigated with normal saline and Betadine to remove debris and bacteria. Hemostasis was achieved, and an absorbable hemostatic agent (Surgicel; Ethicon, Johnson & Johnson, NJ, USA) was placed in the sinus cavity to control bleeding and support tissue regeneration. The OAF was closed using a buccal advancement flap secured with 3-0 Mersilk sutures (Ethicon, Johnson & Johnson, New Brunswick, NJ, USA) in a horizontal mattress pattern for tension-free closure. A pressure pack was applied to reduce the edema and stabilize the flap (Figure 5).

Postoperative care included a seven-day course of broad-spectrum antibiotics and nasal decongestants to manage infection and sinus congestion, respectively. The patient was monitored for complications such as bleeding, infection, or flap dehiscence. The sutures were removed on postoperative day 10. Follow-up evaluations at one week, two weeks, and one month showed uneventful healing with complete fistula closure; no nasal regurgitation, pain, or recurrent discharge; and restored oral function. The patient’s diabetes and hypertension were managed collaboratively with her primary care provider to optimize long-term outcomes. A multidisciplinary approach combining surgical intervention, histopathological confirmation, and medical management ensured the successful resolution of this complex OAF secondary to chronic osteomyelitis in our patient.

## Discussion

The management of OAF secondary to chronic osteomyelitis, as presented in the case of a 50-year-old woman, highlights the complexity of diagnosing and treating this condition, particularly when it is compounded by comorbidities such as uncontrolled diabetes mellitus and hypertension. OAF is a pathological communication between the oral cavity and maxillary sinus, often resulting from dental extractions, infections, trauma, or, as in this case, chronic osteomyelitis [1,3]. The patient’s presentation of persistent swelling, purulent discharge, and oroantral communication, coupled with radiographic evidence of osteolytic lesions and buccal bone necrosis, underscores the destructive potential of osteomyelitis in the maxilla and its role in OAF formation [3]. This case is notable for its rarity, as chronic osteomyelitis leading to OAF is less frequently reported than OAF caused by dental extractions or trauma and poses unique diagnostic and therapeutic challenges.

Chronic osteomyelitis, characterized by persistent bone inflammation and necrosis, is an uncommon but severe cause of OAF [3,5]. The condition is often associated with predisposing factors, such as immunocompromised states, including diabetes mellitus, which impair healing and increase susceptibility to infection [11]. In this case, poorly controlled diabetes likely exacerbated the chronicity of the infection, promoting bone necrosis and sinus involvement. A previous case report by Irie et al. [11] described an acute exacerbation of chronic osteomyelitis in a man with uncontrolled type 2 diabetes mellitus, emphasizing the role of impaired immune responses in disease progression. This rarity underscores the importance of considering osteomyelitis in the differential diagnosis of OAF, particularly in patients with systemic risk factors for osteomyelitis.

The etiology of OAF in this case aligns with that reported in the literature, where chronic infections, particularly in the posterior maxilla, can erode the thin sinus floor and create a fistula [1,3,11]. The proximity of the maxillary molars to the sinus, combined with hypertrophic sinus lining and bone necrosis, facilitates the bidirectional exchange of oral bacteria and nasal secretions, perpetuating inflammation [3]; 50%-60% of untreated OAFs progress to chronic sinusitis, often with mucosal thickening, as observed in our patient’s CBCT findings [12]. The extensive osteolytic lesion and sinus wall erosion observed in this case further differentiate it from typical OAF presentations, which are often limited to smaller defects after tooth extraction [1]. Histopathological confirmation of inflammatory cell infiltration, bone necrosis, and hypertrophic sinus mucosa provided definitive evidence of osteomyelitis as the underlying cause, ruling out other differentials such as neoplasms or fungal sinusitis [3].

The diagnostic process in this case was comprehensive, utilizing CBCT to delineate the extent of bone destruction and sinus pathology, consistent with the recommendations of Donald and Nayak [7], for its superior visualization and lower radiation exposure compared to conventional CT. The initial misdiagnosis of nasal polyps and chronic sinusitis by an ENT specialist treated with FESS highlights a common diagnostic pitfall in atypical OAF presentations. The persistence of symptoms post-FESS necessitates a multidisciplinary approach to address the underlying osteomyelitis and fistula.

The surgical approach involved the extraction of the affected teeth, debridement of the necrotic bone, excision of the hypertrophic sinus lining, and closure with a buccal advancement flap, consistent with established protocols for OAF repair [13]. The use of a hemostatic agent and broad-spectrum antibiotics addressed the infectious and hemorrhagic risks, particularly given the patient’s diabetes status [14]. A previous case report by Huh et al. [15] described a similar case of chronic osteomyelitis with chronic sinusitis in a female patient with uncontrolled type 2 diabetes mellitus who underwent widespread sequestrectomy of the lesion, followed by defect coverage using a buccal fat pad flap. The complete resolution of symptoms and radiographic evidence of bone remodeling at the one-month follow-up in our case affirmed the efficacy of this tailored approach, supported by the collaborative medical management of the patient’s diabetes and hypertension.

This case differs from previous case reports on the primary etiology of chronic osteomyelitis without preceding dental trauma or extractions, emphasizing the role of systemic comorbidities in its progression. The absence of prior dental interventions and the extensive bone destruction observed radiographically highlight the aggressive nature of osteomyelitis-driven OAF, necessitating thorough debridement and sinus mucosal excision to prevent recurrence. A multidisciplinary approach that integrates surgical intervention with medical optimization is critical for a successful outcome.

This case report is limited by its single-case nature, which restricts generalizability. The lack of detailed documentation on patients’ hypertension management and compliance may have overlooked additional factors. Long-term follow-up beyond one month has not been reported, limiting insights into potential recurrence or late-stage complications. Additionally, microbiological analysis of the necrotic tissue was not performed, which could have identified specific pathogens to guide the antibiotic therapy. Despite these limitations, this case underscores the importance of considering chronic osteomyelitis in atypical OAF presentations and highlights the efficacy of comprehensive surgical and medical management in achieving favorable outcomes in such cases.

## Conclusions

This case report describes the successful management of a rare OAF caused by chronic osteomyelitis in a 50-year-old woman with systemic comorbidities. Persistent swelling, purulent discharge, and osteolytic lesions were assessed using CBCT-guided diagnosis and histopathology to confirm the diagnosis of osteomyelitis-driven OAF. Surgical intervention, including tooth extraction, necrotic bone debridement, sinus lining excision, and buccal flap closure, combined with antibiotics and medical optimization, achieved complete symptom resolution and bone remodeling by the one-month follow-up.
